# Oral Administration of GnRH and Domperidone via Gel Feed and Their Effect on Reproductive Performance of *Devario devario* (Bengal Danio), an Ornamental Fish

**DOI:** 10.3390/gels11070554

**Published:** 2025-07-18

**Authors:** Suparna Deb, Pradyut Biswas, Soibam Khogen Singh, Gusheinzed Waikhom, Reshmi Debbarma, Shubham Kashyap, Jham Lal, Khusbu Samal, Supratim Malla, Nitesh Kumar Yadav, Ng. Chinglembi Devi, Pronob Das, N. Sureshchandra Singh, G. Deepak Reddy, Surajkumar Irungbam

**Affiliations:** 1 College of Fisheries, Central Agricultural University, Lembucherra, Agartala 799210, Tripura, India; 2 Krishi Vigyan Kendra, Ukhrul, ICAR Research Complex for NEH Region, Imphal 795142, Manipur, India; 3 ICAR-Central Inland Fisheries Research Institute, Regional Centre, Guwahati 781006, Assam, India

**Keywords:** gonadosomatic index, fecundity, GSI, *Devario devario*

## Abstract

This study investigated the effects of dietary Gonadotropin-releasing hormone (GnRH) and domperidone on the reproductive performance of *Devario devario* during a 40-day trial. Five treatment groups received varying doses of GnRH (100, 50, 25, 12.5 µg/kg body weight) in combination with domperidone (50, 25, 12.5, 6.25 mg/kg body weight), embossed in a gel-based diet alongside a control group without the exogenous hormones. Reproductive performance was examined by measuring the gonadosomatic index, fecundity, reproductive hormone levels, and histological features of the gonads, blood parameters, and antioxidant enzyme activity. The T1 group (100 µg GnRH + 50 mg domperidone) exhibited the highest GSI in both sexes. The histological analysis of testes from T1, T2 (50 µg GnRH + 25 mg domperidone), and T3 (25 µg GnRH + 12.5 mg domperidone) groups revealed an increased presence of late-stage spermatids and spermatozoa. In females, the T2 group produced the highest proportion of advanced-stage oocytes and demonstrated the greatest absolute fecundity (1300 ± 23 eggs). However, the control group showed the highest fertilization and hatching rates. Testosterone levels were significantly elevated in the T3 group, while vitellogenin levels increased in the T1 and T2 groups. Antioxidant enzyme activity varied, with the T1 group displaying higher superoxide dismutase activity in gills and liver, and the T2 group showing increased SOD activity in muscle and brain. Improvements in haematological parameters were observed across all treatments. These results suggest that an optimal dose of 50 µg GnRH + 25 mg domperidone can enhance reproductive performance in *D. devario*.

## 1. Introduction

Captive fish breeding programs frequently encounter difficulties in stimulating reproductive maturation and achieving successful spawning. While environmental manipulation techniques, such as adjusting photoperiod and temperature, can be effective for some species [1,2,3], hormonal induction remains a crucial tool, especially when natural spawning cues are insufficient or species-specific reproductive requirements are poorly understood [4,5,6]. This is because the hypothalamic–pituitary–gonadal axis (HPG axis), responsible for regulating reproductive development and gametogenesis, can be directly stimulated through hormonal manipulation. Male fish in captivity may produce less milt and have lower-quality sperm, while female fish frequently show restrictions in ovarian development, oocyte maturation, ovulation, and spawning.

Gonadotropin-releasing hormone and dopamine play significant roles in regulating the reproductive system in fish. The follicle-stimulating hormone and the luteinizing hormone, which are necessary for gonadal development and function, are produced in response to GnRH action on the pituitary gland [7,8]. The follicle-stimulating hormone (FSH) is crucial for sexual development and reproduction, influencing the activity of both ovaries and testes. However, under natural conditions, dopamine exerts an inhibitory effect on GnRH, creating a feedback loop that modulates gonadotropin release. This dopamine-mediated suppression can limit the effectiveness of exogenous GnRH administration in inducing reproductive maturation. To overcome this, dopamine antagonists, such as domperidone (DOM), are often used in conjunction with GnRH. By blocking the inhibitory effects dopamine, DOM enhances the efficacy of GnRH, leading to improved reproductive outcomes. Several studies have shown the effectiveness of combined GnRH and domperidone treatment in promoting gonad development in various fish species, including ornamental and commercially important species [9].

The oral administration of hormones through feed offers a less stressful and more effective alternative to traditional methods like injections or implants, which can induce stress in broodstock that leads to mortality, poor oocyte quality, and reduced larval survival [10]. Gel feeds offer a versatile platform for delivering not only hormones but also flavour enhancers, attractants, nutritional supplements, medications (including antibiotics and chemotherapeutics), and microbial supplements like probiotics [11,12]. Such a delivery method minimizes handling stress and allows for controlled hormone absorption from the intestine into the bloodstream [12]. Their ease of use, particularly in captive conditions where they can be placed in feeders, further enhances their wider applications for aquaculture.

India, with its rich fish biodiversity, plays a significant role in the global ornamental fish trade, which is considered a multi-billion-dollar industry [13]. This industry involves over two billion live fish sold annually, with a global market value exceeding USD 15 billion. While European countries and Japan represent major markets, over 65% of these exports originate from Asia [14], highlighting the region’s significance in ornamental fish production. The northeastern region, which is one of the two biodiversity hotspots in India that contribute significantly to the ornamental fish trade, accounts for approximately 85% of the industry [15]. Bengal danio (*Devario devario*), a cyprinid fish inhabiting slow-moving streams and lentic water bodies is one of the most expensive fish on global market. However, the present trade, which relies on wild-caught specimens, haschallenges due to unpredictable seed availability and potential ecological impacts [16]. Thus, it is essential to create sustainable breeding methods for species like the Bengal danio in order to satisfy consumer demand and guarantee the long-term preservation of the fish.

Given that hormonal induction techniques hold promise for enhancing reproductive activity in fish, the oral delivery of gonadotropin-releasing hormone and domperidone remains unexplored as a strategy for artificial reproduction in fish. To remedy this, we used our previously designed gel diet [12] as a medium for the effective carriage of the hormones in the gastro-intestinal tract. The objective of this study wasto examine the effectiveness of varying doses of GnRH and domperidone, delivered via a novel gel-based diet, on the reproductive performance and stress physiology of Bengal danio. The study encompassed both laboratory and outdoor trials to evaluate the feasibility and effectiveness of this innovative hormone delivery method for promoting early maturation and enhancing reproductive output in this commercially important species.

## 2. Results and Discussion

### 2.1. Reproductive Performance

The effects of treatments on gonadosomatic index in *D. devario* are presented in Table 1 and Table 2 for females and males, respectively. Initially, no notable variations in GSI were noted between treatment groups in either sex. By day 20, female GSI was significantly higher in the T1 group compared to the T4 and control groups, with no significant differences between T2, T3, and the control. A similar pattern was observed in males at day 20, with T1 and T2 exhibiting significantly higher GSIs compared to T4 and the control. At day 40, female GSI was significantly higher in the T2 group compared to the T4 and control groups, while male GSI was significantly higher in the T1, T2, and T3 groups compared to the T4 and control groups. The mean weight of fish during stocking was 0.88 ± 0.05 g.

### 2.2. Histological Gonad Changes

**Figure 1:**
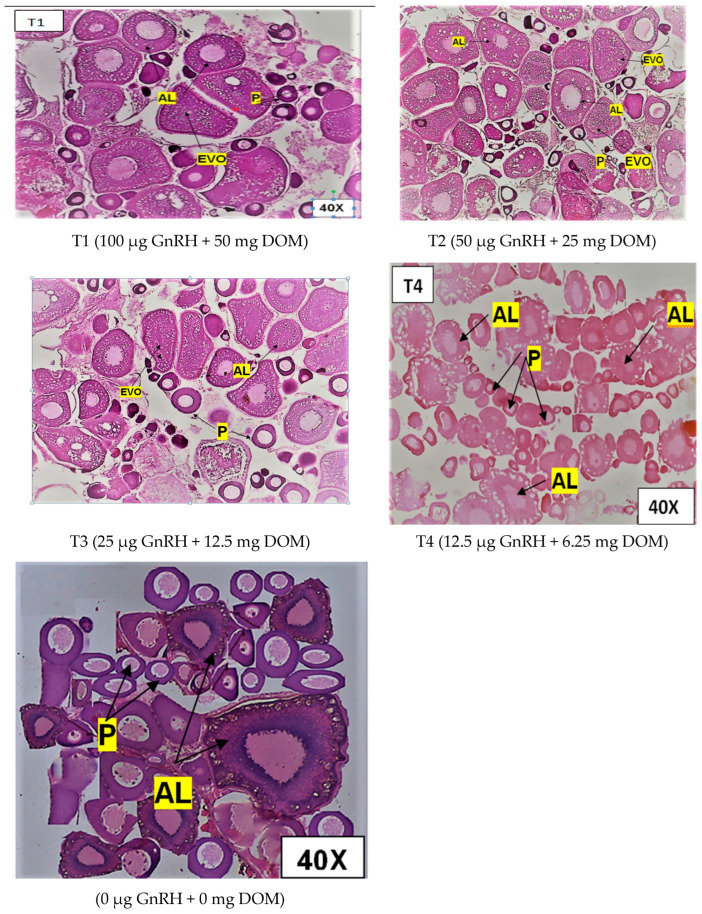
Photomicrographsof H&E-stained ovary of *D. devario* (female) fed gel diets supplemented with different levels of GnRH and DOM. Maturation stages in treatment groups are indicated in the micrographs T1–T4. P—perinuclear cell; AL—alveolar oocyte; EVO—early vitellogenic oocyte. Magnification at 40×.

**Figure 2:**
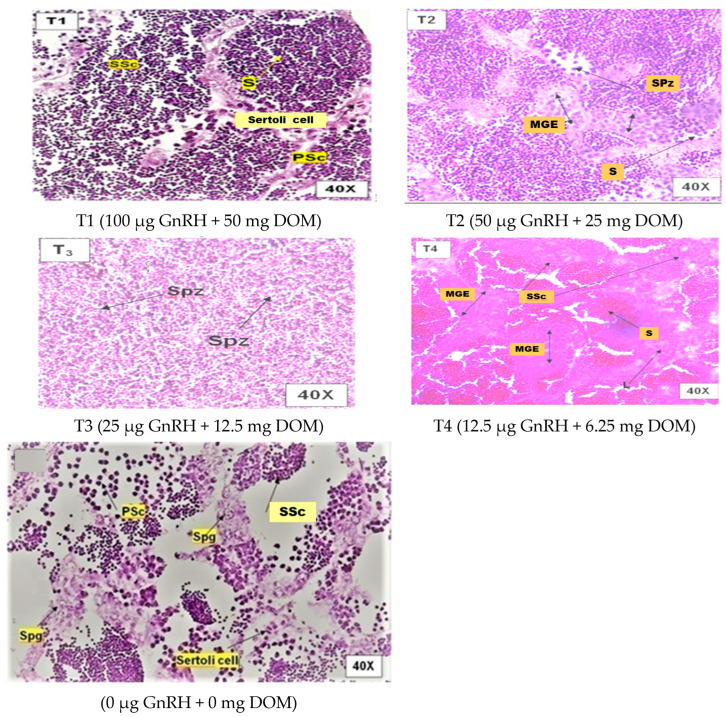
Photomicrographsof H&E-stained testis of *D. devario* (male) fed gel diets supplemented with different level of GnRH and DOM. Maturation stages in treatment groups. SSc—secondary spermatocytes; S—spermatid; PSc—primary spermatocyte; Spz—spermatozoa; L—Leydig cells; Spg—spermatogonia. Magnification at 40×.

Figure 1 illustrates the influence of dietary GnRH and DOM supplementation on ovarian maturation in *D. devario*. Treatment T2 (50 μg GnRH and 25 mg DOM) resulted in the highest percentage of advanced stage oocytes, characterized by prominent vitellogenic and alveolar oocytes. Conversely, treatments T1 (100 μg GnRH and 50 mg DOM) and T3 (25 μg GnRH and 12.5 mg DOM) exhibited a marked increase in the proportion of atretic oocytes compared to other treatments. The control group (C; 0 μg GnRH and 0 mg DOM) and T4 (12.5 μg GnRH and 6.25 mg DOM) displayed a predominance of early-stage oocytes, with no significant differences in ovarian maturity observed between these two groups (*p* > 0.05). Figure 2 depicts the effects of dietary GnRH and DOM supplementation on testicular maturation in *D. devario*. Histological analysis revealed a significantly greater abundance of late-stage spermatids and spermatozoa in the testes of fish receiving T1 (100 μg GnRH and 50 mg DOM), T2 (50 μg GnRH and 25 mg DOM), or T3 (25 μg GnRH and 12.5 mg DOM) treatments compared to controls. In contrast, the control and T4 (12.5 μg GnRH and 6.25 mg DOM) groups exhibited a predominance of spermatogonia and spermatocytes, indicating earlier stages of spermatogenesis.

### 2.3. Breeding Performance

Table 3 presents the effects of treatments on reproductive parameters in *D. devario*. Absolute fecundity was significantly higher in T2 (1300 ± 23 eggs) compared to all other treatments (*p* < 0.05), while T1 and T3 exhibited moderately higher fecundity than T4 and the control group. Conversely, fertilization rate was significantly higher in the control and T4 groups (86.0 ± 0.58% and 85.3 ± 0.88%, respectively)compared to all other treatments (*p* < 0.05). Hatching success was highest in the control group, although this difference was only statistically significant compared to T1 and T2 (*p* < 0.05). No significant differences in larval survival were observed between any of the treatment groups (*p* > 0.05).

### 2.4. Sperm Quality and Quantity

Table 4 presents the effects of treatments on sperm parameters in *D. devario*. Sperm volume was significantly higher in the T1, T2, and T3 treatment groups compared to the T4 and control groups (*p* < 0.05). Sperm concentration also differed significantly between treatments, with the highest concentration observed in T3 and the lowest in the control group (*p* < 0.05). No significant differences in sperm motility were observed between any of the treatment groups (*p* > 0.05).

### 2.5. Stress Hormones

The effects of GnRH and DOM supplementation on liver and muscle cortisol levels in *D. devario* are presented in Table 5. At day 20, liver cortisol was significantly elevated in the T1 (100 µg GnRH/kg + 50 mg DOM/kg feed) group compared to all other treatments (*p* < 0.05). By day 40, both T1 and T2 (50 µg GnRH/kg + 25 mg DOM/kg feed) exhibited significantly higher liver cortisol levels compared to other groups (*p* < 0.05). Muscle cortisol levels followed a similar trend, with T1 displaying significantly higher levels than all other treatments at both day 20 and day 40 (*p* < 0.05). T2 also showed elevated muscle cortisol compared to the lower dose treatments and control at both time points.

Table 6 presents the effects of GnRH and DOM supplementation on liver and muscle glucose levels in *D. devario*. At day 20, liver glucose was significantly higher in the T1 group (100 µg GnRH/kg + 50 mg DOM/kg feed) compared to all other treatments (*p* < 0.05). A similar pattern was observed at day 40, with both T1 and T2 (50 µg GnRH/kg + 25 mg DOM/kg feed) exhibiting significantly elevated liver glucose levels compared to other groups (*p* < 0.05). Muscle glucose levels were also significantly higher in T1 and T2 compared to other treatments at day 20. By day 40, muscle glucose remained elevated in the T1, T2, and T3 (25 µg GnRH/kg + 12.5 mg DOM/kg feed) groups compared to the T4 (12.5 µg GnRH/kg + 6.25 mg DOM/kg feed) and control groups (*p* < 0.05).

### 2.6. Antioxidant Enzymes Status

Effect of GnRH and DOM supplementation on antioxidant enzymes of *D. devario* are provided in Table 7. Result shows that, SOD level in brain was observed to be no significant difference among the treatment and control groups (*p* > 0.05). The values were observed to be significantly higher in treatment groups, especially in T2 94.2 ± 2.45 mg protein^−1^ and 34.4 ± 2.04 mg protein^−1^ in the gill and liver, respectively, compared to others (*p* < 0.05). In muscle, SOD level was recorded to be significantly higher in T2 but there were no differencesbetweenT1 and T3; the lowest values were observed in T4 and the control (*p* < 0.05). In brains, the CAT level was higher in T2 treatment 281 ± 24.1 mg protein^−1^, whereas the control exhibited the lowest value. Significant differences were observed between the treatment groups and the control group (*p* < 0.05). The values were observed to be significantly higher in treatment groups, especially in T2 at 281 ± 19.8 mg protein^−1^, and the lowest values were observed in T3, T4, and the control group (*p* < 0.05) in gills. In the case of 50 µg kg^−1^ BW, the supplemented dose resulted ina higher value than the others, and the control exhibits the lowest value (*p* < 0.05). In muscle, the CAT level was recorded to be significantly higher in T2, but there were no differences between T1, T3, and T4; the lowest values were observed in T4 and control (*p* < 0.05).

### 2.7. Levels of Sex Hormones

Table 8 shows the effects of treatments on testosterone and vitellogenin levels in *D. devario*. Testosterone levels were significantly higher in the T3 group compared to in all other treatment groups (*p* < 0.05). While T1 and T2 exhibited similar testosterone levels, both were significantly higher than that of the control group. Vitellogenin levels were significantly elevated in the T1 and T2 groups compared to in the control group (*p* < 0.05).

### 2.8. Discussion

#### 2.8.1. Gonadosomatic Index (GSI)

The gonadosomatic index (GSI), defined as the ratio of gonad weight to body weight, is a crucial indicator for assessing gonadal growth, the timing of maturation and spawning [17], and fish sexual maturity as it reflects the relative weight of gonads (ovaries and testes) in relation to body weight [18]. GnRH is crucial in regulating the hypothalamic–pituitary–gonadal (HPG) axis, which is essential for reproductive functions in fish. Studies have shown that GnRH stimulates the release of gonadotropins from the pituitary gland, promoting follicular development and oocyte maturation [19]. Specifically, research indicates that GnRH influences the expression of kisspeptin, a key regulator of reproductive hormones, in zebrafish brains, thereby playing a significant role in reproductive timing and success [20]. Domperidone, a dopamine antagonist, has been shown to mitigate stress-induced reproductive suppression. In a study involving stressed mosquitofish, treatment with domperidone resulted in increased follicular development and pregnancy rates [21]. This was associated with enhanced GnRH immunolabeling in treated fish compared to those only subjected to stress. The findings suggest that dopamine may inhibit GnRH activity during stress, and that domperidone can counteract this effect by restoring GnRH signalling pathways [22]. Similarly results find in the present study, GnRH stimulates the development of GSI in the Bengal danio. In cases of female fish exhibiting a higher GSI after 20 days of supplementation with increasing doses of GnRH and DOM, the highest GSI was initially observed in the group receiving the highest doses. However, by 40th day, the group receiving a lower supplementation level showed the highest GSI. A similar trend was observed in male fish, with the highest GSI initially found in the group receiving the highest supplementation; however, this effect shifted by 40th day, favouring the lower supplementation group. These findings suggest that GnRH, working synergistically with DOM, directly stimulates gonadotropin activity through the hypothalamic–pituitary–gonadal axis, thereby enhancing the vitellogenesis process. Some other researchers reported the GnRHa administration promotes germ cell proliferation and spermatozoid differentiation in various fish species, including zebrafish, golden rabbit fish, Senegalese sole, and yellow catfish [23,24,25,26,27].

#### 2.8.2. Ovarian Maturation of Bengal Danio

Gonadotropin-releasing hormone (GnRH) and domperidone are critical components in the regulation of ovarian maturation across various species, particularly in fish. Their combined application, often referred to as Ovaprim, has been studied for its effectiveness in inducing oocyte maturation and ovulation. The primary hormone that causes the pituitary gland to release gonadotropins, which in turn encourages ovarian development and maturation, is GnRH. Domperidone, a dopamine D2 receptor antagonist, enhances the function of GnRH bysuppressing the inhibiting effects of dopamine on gonadotropin release. This synergistic effect is particularly important in species where natural reproductive processes may be disrupted or need to be artificially induced. The authors of [28] reported that the combination of GnRH and domperidone effectively induces oocyte maturation in various fish species. For instance, research indicated that intramuscular injections of Ovaprim resulted in the significant maturation and ovulation of oocytes in species such as *Triakisscyllium* and *Triaenodon obesus*. In another study, the administration of GnRH agonists along with domperidone led to observable vitellogenesis and ovulation within a defined timeframe, demonstrating their role in facilitating ovarian development [29].

Fish oocyte maturation occurs through two meiotic divisions, during which the oocyte produces two polar bodies. A nucleus of the primary oocyte enlarges throughout this process, is arrested at the diplotene stage, and shifts to a more peripheral position. Its membrane degrades, and a polar body is extruded to complete the first meiotic division [30]. Oocyte nuclear envelope disintegration or germinal vesicle disintegration (GVBD) at the prophase/metaphase transition is commonly seen as proof of oocyte maturation progress [31,32]. Following that, the second meiotic division begins but only continues to the metaphase stage. During these events, an animal pole separates from the vegetal pole. The yolk also matures and becomes less opaque as a result of hydration and maturation [33]. Vitellogenesis is followed by significant oocyte development due to the ingestion of yolk precursor proteins, primarily vitellogenin (Vtg), and maybe extremely low-density lipoproteins [34,35].

In the present study, histological analysis observed the highest number of early vitellogenic oocytes in the 50 µg GnRH kg^−1^ + 25 mg DOM kg^−1^ dietary intake group, which was significantly higher than in the remaining treatment groups. In addition, the number of early vitellogenic oocytes was very low, but the number of alveolar oocytes and peri-nucleolar oocytes was higher in treatment group 2. Moreover, other researchers have reported similar findings in *Osteochilus melanopleurus* [36], goldfish [25], and whitefish [36]. This could be due to an increase in hormone levels in fish bodies, which shortens the time it takes for an ovum to reach the vitellogenic stage. Chronic-release implants containing GnRHa, either alone or in conjunction with testosterone or E2, have previously been demonstrated to increase oocyte growth in striped mullet [37], as well as in Mediterranean grey mullet [38,39]. The purpose of these treatments was to increase the production of gonadotropins by inducing steroidal positive feedback in the pituitary glands of young fish [40]. The prolonged administration of GnRHa was expected to stimulate pituitary gonadotropin release [41].

#### 2.8.3. Effect on Testicular Maturation

GnRH is essential for inducing the pituitary gland to release the luteinizing hormone (LH) and the follicle-stimulating hormone (FSH), which in turn encourage the synthesis of testosterone in males. This cascade is vital for sexual maturation and fertility [42]. Studies have shown that multiple injections of GnRH analogues can accelerate testicular development and maturation, demonstrating its effectiveness in enhancing spermatogenesis [43]. Domperidone is a dopamine antagonist that primarily influences prolactin levels. Elevated prolactin can inhibit testosterone production, leading to reduced testicular function. Research indicates that the chronic administration of domperidone can lead to decreased serum testosterone levels and impaired sexual behaviour in male rats, suggesting a negative impact on testicular maturation [44]. In the current study, the histological examination of testes demonstrated that the dietary supplementation of GnRH and domperidone enhanced the number of spermatids by more than 25 µg kg^−1^ feed and 12.5 mg kg^−1^ feed, respectively (T3 group). The control group had an abundance of primary and secondary spermatocytes, spermatogonia cells, and Leydig cells, indicating that the testicular event was immature. In extreme situations, an increased number of germinal epithelial cells and spermatocytes were found (T1 and T4, respectively). These groups also had fewer spermatids and Sertoli cells. Similar results were found in common carp [45]. The control testes exhibited all stages of germ cell development, mostly spermatocytes and spermatids, with a small number of spermatozoa, according to the histological analysis. Interestingly, testes from Ovatide^®^-treated fish showed morphological variations across treatments, with 25 µg kg^−1^ GnRH and 12.5 mg kg^−1^ DOM supplementation resulting in clear evidence of spermiation and lobules loaded with a considerable number of spermatozoa. This could be because the amount is less effective at inducing gonadal development [46]. The authors of [47] reported that the administration of synthetic GnRH alongside domperidone (as part of Ovaprim) has been shown to enhance oocyte maturation in carp. It seems that this impact is mediated by the reduction in oxidative stress and the stimulation of maturation-promoting factors (MPF). However, the relationship between these substances is complex. While GnRH can stimulate testicular maturation, excessive prolactin due to domperidone may counteract some of these effects by inhibiting testosterone production. Therefore, the timing and dosage of these treatments are critical for optimizing their benefits in reproductive health.

#### 2.8.4. Effect on Reproductive Hormones

Gonadotropin-releasing hormone (GnRH) and its analogues, alongside dopamine antagonists like domperidone, play significant roles in regulating fish reproduction. GnRH is a key neuropeptide that promotes gonadotropin release from the pituitary gland, which is essential for oocyte maturation and ovulation. The implantation of GnRH agonists (GnRHa) can induce vitellogenesis and ovulation in various fish species. For instance, in red seabream, both GnRHa and its combination with domperidone resulted in significant ovarian development, with vitellogenesis observed as early as Day 10 and ovulation observed by Day 20 after treatment [29]. In the present study, the reproductive hormone testosterone was found to be higher in the T3 group, which indicates improved reproductive advancement in males. Similarly, Ref. [28] reported the role of Synthetic Salmon GnRH and Domperidone (Ovaprim^®^) in sharks (*Triakisscyllium*, *Balantiocheilosmelanopterus*, and *Triaenodon obesus*), associated with testosterone levels in blood. Vitellogenin is a protein found in the blood of female fish during maturation as a result of vitellogenesis [48] that can accurately determine the fish’s reproductive condition. The administration of GnRHa has been associated with increased levels of serum luteinizing hormone (LH), testosterone, and estradiol-17beta, which are crucial for reproductive processes. The expression of gonadotropin subunit genes also shows up-regulation following GnRHa treatment, further supporting its role in enhancing reproductive hormone production [19]. Domperidone acts as a dopamine D2 receptor antagonist, counteracting the inhibitory effects of dopamine on gonadotropin release. Dopamine typically suppresses gonadotropin secretion; hence, using domperidone can enhance the effectiveness of GnRH treatments. In stressed mosquitofish, for example, domperidone treatment increased follicular development and pregnancy rates by promoting the recovery of GnRH-immunoreactive fibres in the pituitary gland [21]. This suggests that domperidone can mitigate stress-induced reproductive suppression.

#### 2.8.5. Effect on Sperm Parameters

Gonadotropin-releasing hormone (GnRH) and its analogues, particularly when combined with dopamine antagonists like domperidone, have significant effects on fish sperm parameters. The use of these hormones is crucial in aquaculture for inducing spermiation and enhancing sperm quality. GnRH causes the pituitary gland to release gonadotropins, which in turn promotes gamete maturation and release in fish. This process is essential for successful reproduction in controlled environments [28,49]. As a dopamine D2 receptor antagonist, domperidone counteracts the inhibitory effects of dopamine on GnRH release, facilitating higher levels of gonadotropins and promoting spermiation [28]. In the present study, the highest sperm concentration and motility was observed in the treatments groups compared to the control group. These could be the result of an LH increase after hormone therapy. Similarly, Ref. [50] reported that GnRH and domperidone increased the sperm motility in *Osmerus eperlanus* L. GnRH resulted in increased spermiation in sea bass by inducing gonadotropin activity [51]. In this study, we observed that fish fed 25 µgGnRHa kg^−1^ with 12.5 mg Dom kg^−1^ diets showed the maximum sperm concentration compared to other treatments groups. This could be for the same reason that embedding pellets of GnRHa did not impact sperm activity in greenback flounder (*Rhombosoleatapirina*), as [52] discovered. Clearwater and Crim (1998) also reported that the GnRHa therapy increased sperm motility in yellowtail flounder [53] and increased spermatozoa motility in cyprinids [54]. Hormonal treatments using GnRH analogues combined with domperidone (e.g., Ovaprim) have been shown to significantly increase sperm density and total sperm volume in various fish species. For instance, studies indicate that Ovaprim treatment resulted in a marked increase in total milt volume compared to control groups [49]. The quality of sperm motility is critical for fertilization success. Research has demonstrated that treatments with GnRH analogues can enhance motility parameters, including the velocity and percentage of motile sperm. For example, higher motility percentages were observed 12 hafter treatment with Ovaprim [55,56]. In this study, a notable reduction in fertilization rate in the hormone-treated group was observed compared to in control fishes. As such, several authors reported improvement in fertilization rate after hormonal treatment; however, we speculate that interplay of factors, such as disruptions to the HPG axis, impaired gamete quality, oxidative stress, and epigenetic modifications, might also affect the rate of fertilization.

#### 2.8.6. Effect on Antioxidant Enzyme Activity

Stress is known to have an impact on a variety of physiological systems, including fish reproduction [57,58]. Although stressor exposure during aquaculture operations has been proven to alter reproduction in most species, the effects are ambiguous. Cells produces reactive oxygen species (ROS) during stress, which causes cell death or organ damage. This imbalance is known as oxidative stress [59]. Superoxide dismutase (SOD) and catalase (CAT) [60,61]. The current study found a constant increase in antioxidant enzyme activity, implying defective antioxidant defence systems as a result of the excess production of superoxide radicals caused by the administration of dietary GnRH and domperidone [6]. Free radicals produced during egg development and ovulations have been linked to oxidative stress [31,62]. In the current investigation, we found that T2 had higher SOD and CAT activity than the other treatment groups, indicating oocyte maturation.

#### 2.8.7. Effect on Breeding Performance of Fish

The supply of high-quality fish seeds in sufficient and reliable amounts is one of the production requirements that must be fulfilled in efforts to advance fish farming [63]. GnRH causes the pituitary gland to release more gonadotropins, which are crucial for oocyte maturation and ovulation. However, dopamine can inhibit this process. Domperidone functions as an antagonist of the dopamine D2 receptor, thereby negating this inhibitory effect and facilitating higher gonadotropin release when used alongside GnRH [4,28]. In the present study, T2 had the highest absolute fecundity (50 µg GnRH kg^−1^ feed with 25 mg DOM kg^−1^ feed). Similarly, Ref. [4] reported the GnRH analogues (GnRHa) have been effectively used to induce spawning and improve egg production in various fish species, including common carp and catfish. A neuropeptide called GnRH causes the pituitary gland to release more gonadotropins, which are essential for fish ovulation and gamete development.

In the present study, the highest hatching rate was observed in the T2 groups compared to the other groups, while control group showed the lowest hatching percentages. According to [64], FSH activity in the pituitary drives the release of oestrogen in the follicle, which influences follicular growth. According to [65], sGnRH + domperidone not only induces broodstock to ovulatebut is also related to fertilization, as increasing the hormone dose above its optimum level reduces the hatching rate. It is reported that fish tissues, including the gonads, can experience oxidative stress due to high hormone dosages. Gamete viability and embryonic development can be impacted by oxidative stress [66]. It is therefore suggested that comprehensive dose–response studies should be carried out to determine optimal hormone doses for key species. However, unless there are environmental factors that affect the hatching process, like abrupt changes in temperature, oxygen, and pH, the percentage of egg hatchability is always determined by the percentage of egg fertilization, and the higher the percentage of egg fertilization, the higher the percentage of egg hatchability [67]. Larval survival is impacted by environmental factors, including as disease, stocking density, and water quality, but hormone injection has no direct effect on it. Both biotic and abiotic factors influence the survival rate of fish [68].

## 3. Conclusions

The incorporation of oral GnRH- and domperidone-supplemented gel-based feed into the breeding regimen of Bengal danios is a potential technique for improving reproductive success. In captive conditions, spawning failure occurs due to a lack of appropriate environmental conditions for the development of ovaries or spawning. Future work should align the molecular basis to undermine the physiological pathways and alterations in the gene transcripts by applying modern omics tools. This approach not only increases spawning and fertility but also improves GSI, fertilization, and hatching rate for promoting sustainable aquaculture practices. Future studies should focus on improving hormone dosage and timing in order to further enhance these strategies and ensure their effectiveness on a broader scale inside aquaculture systems. Alternate-day feeding, enabled by the stability of the prepared gels and optimized treatment duration, offers a potential strategy to improve the efficiency of the present experimental design. The commercial application of gel feed, though still in its infancy, holds great potential. Industry collaboration with research findings could unlock specific-purpose applications, such as the delivery of hormones, offering both improved water quality and targeted delivery.

## 4. Materials and Methods

### 4.1. Animal Ethics

Experimental fish were handled and raised according to Indian laws. The study protocol and experimental endpoints follow the CPCSEA’s guidelines on animal care and use in scientific research. The Institutional Ethics Committee (IAEC) of the College of Fisheries, Central Agricultural University (I), Tripura, India, approved the study (approval no. CAU-CF/48/IAEC/2018/096, 22 October 2022). Animals were maintained in well-aerated tanks and euthanized with 500 mg/L powdered MS-222 during transport and for experimental completion.

### 4.2. Experimental Fish and Husbandry

The experimental fish *D. devario* were captured from the Khowai River in Tripura and housed in the indoor tanks at the wet laboratory of the College of Fisheries, Central Agricultural University, Lembucherra, Tripura, India. Aquariums were filled with underground water that had been processed and was continuously aerated. Prior to this, standard pre-stocking management procedures such as washing, cleaning, drying, and filling were followed. In order to acclimatize the fish to the experimental setting, they were fed a standard practical diet for 15 days prior to receiving their allocated treatments. Under controlled conditions, an experiment was conducted to investigate the effect of the oral administration of synthetic Salmon GnRH and domperidone (DOM) on the reproductive performance of *D. devario*. In this experiment, a total of 15 aquariums with a depth of 0.4 m were employed, each containing three replicates for each treatment.

### 4.3. Formulation of Feed

In this experiment, we used gel feed to deliver GnRH and DOM. Four experimental diets (gel feed) and one control diet were designed for this purpose (Table 9 and Table 10). The amount of feed required for 40 days to make up the primary diet for each treatment was calculated based on the biomass of the fish in each aquarium. GnRHa and DOM were incorporated into a gel feed base using a blending machine, ensuring homogenous distribution. The detailed gel feed preparation protocol, established in our laboratory, is described in [12]. The mixture was then covered in aluminium foil and plastic wrapper to prevent water absorption. The wrapped-up content was then placed in a hot water bath at 40 °C for 2 h. The content was then placed in the refrigerator for the night. Following this, the gel feed wasready to befed to the fish and was stored in a moist-free, refrigerated environment.

Fishes were fed artificial gel feed twice daily at a rate of 5% of their body weight for a period of 40 days. The gel feed was supplemented with varying doses of GnRH + DOM (Sigma, New Delhi, India). To avoid over-conditioning and potential negative effects of continuous hormone exposure, the hormone-enriched gel feed was administered every other day, alternating with a diet of live plankton.

### 4.4. Experimental Design and Set-Up

The study was conducted at the College of Fisheries, Central Agricultural University, India, utilizing a completely randomized design (CRD). Fifteen 40 L aquariums were used, each representing a replicate of one of the experimental diet treatments. Prior to use, bore-well water was aerated for four days in a 1000 L storage tank. Each aquarium was filled with 30 L of this seasoned water and stocked with ten (*n* = 10) Bengal danio (*Devario devario*) that had been acclimated to laboratory conditions. Fish were fasted on the day of transfer. All aquariums were maintained under a 12:12 light/dark photoperiod and equipped with a central blower for continuous aeration.

### 4.5. Water Quality Analysis

The key water quality parameters like pH, temperature, dissolved oxygen (DO), hardness, and alkalinity were examined on fortnightly basis and are presented in Table 11. The standard method for the estimation of water quality parameters described by APHA, 2005 [69] was followed in this study.

### 4.6. Reproductive Performance

The reproductive performance of experimental animals was estimated in terms of reproductive parameters like fecundity and the gonadosomatic index (GSI). The following formulae were used:

$$Total\;fecundity=\frac{\left(No.of\;eggs\;in\;the\;sample\times weight\;of\;the\;gonad\right)}{Weight\;of\;the\;sample}$$

$$Gonadosomatic\;Index\;(GSI)=\frac{Weight\;of\;gonad\;(g)}{Bodyweight\;of\;fish\;(g)}\times 100$$

### 4.7. Sperm Parameters

Males were squeezed, as described above, and sperm from several males was pooled to produce a final volume of at least 8 μL/trial. This sample was diluted to 16,109 cells mL^−1^, measured on a haemocytometer, then portions were diluted with HBSS to yield 108, 107, 106,105, and 104 cells mL^−1^. Sperm motility was established using visual observation with a light microscope (Lieca DM 750, Mumbai, India) at 10× magnification.

### 4.8. Breeding Performance

All the females were examined for ripeness at 10 h post-injection, and these observations continued at 1 h intervals by examining the genital papilla and gently pressing the abdomen with hand and squeezing towards the urogenital opening. Eggs from the ripe females were stripped and collected separately in dry and clean plastic trays. The dry method was used for egg fertilization, where the sperm from a ripe male was added directly by placing the sperm onto the eggs. This was followed by gentle mixing for at least 30–40 s. The fecundity, fertilization rate, and hatching rate were determined using the following equations:

Fecundity = number of eggs in 1 g subsample × total weight stripped eggs

Fertilization rate (%) = (number of fertilized eggs/total number of eggs) × 100

Hatching rate (%) = (number of spawns/number of fertilized eggs) × 100

Survival (%) = (number of hatchlings/total number of spawn) × 100

### 4.9. Reproductive Hormone

Plasma testosterone levels were quantified using a commercial ELISA kit (Bioassay Technology Laboratory, EA0009Ge, Shanghai, China), according to the manufacturer’s instructions. Briefly, samples and standards were added to the pre-coated plate, and colour development was measured spectrophotometrically at 450 nm. Testosterone concentrations in the samples were determined by comparing the optical density readings to a standard curve. Vitellogenin levels in tissue homogenates were determined using a quantitative sandwich ELISA kit specifically designed for laboratory research use. Absorbance was measured at 450 nm, in accordance withthe manufacturer’s protocols.

The vitellogenin analysis was performed using the VG ELISA kit (Fish Vitellogenin ELISA KitMBS010726).

### 4.10. Antioxidant Enzyme Assay

Catalase assay was carried out according to the method of [70]. A total of 50 μL of the tissue homogenate was added to 2.45 mL of phosphate buffer (50 mM, pH 7.0), and the reaction was initiated by adding 1.0 mL of H_2_O_2_ solution. The absorbance was measured at 240 nm at 30 s intervals for two minutes. The enzyme activity was expressed as nmol of H_2_O_2_ decomposed/min/mg protein. The superoxide dismutase activity (SOD) enzyme was assayed according to the method of [71], which is based on the oxidation of the epinephrine–adrenochrometransition by the enzyme. One unit of SOD activity is the amount of protein required for a 50% inhibition of epinephrine auto-oxidation.

### 4.11. Stress Parameters

#### 4.11.1. Cortisol Level Analysis

After collecting liver and muscle samples from freshly died fish, the samples were prepared using maceration in sucrose solution and collected supernatant. With this supernatant, acortisol analysis was performed usingt he ELISA Kit (CALBIOTECH Cortisol ELISA CO368S). The Calbiotech Inc. (El Cajon, CA, USA) cortisol test kit is a solid phase competitive ELISA. The samples, the cortisol–HRP conjugate, and the anti-cortisol-biotin solution were added to the wells, which were coated with streptavidin. Cortisol in the fish homogenate/serum samples was supplemented with the cortisol enzyme (HRP) conjugate for binding sites. The unbound cortisol enzyme conjugate was washed off using a washing buffer. Upon the addition of the substrate, the intensity of colour is inversely proportional to the concentration of the cortisol in the samples. A standard curve was prepared which relates the colour intensity to the concentration of the cortisol.

#### 4.11.2. Glucose Analysis

For the determination of glucose in serum, a glucose diagnostic kit (Coral Clinical Systems, Goa, India) was used and was based on the GOD/POD method [72]. The kit was based on the principle that glucose oxidizes to gluconic acid and hydrogen peroxide in the presence of glucose oxidase. Hydrogen peroxide further reacts with phenol and 4-aminoantipyrine through the catalytic action of peroxidase to form a red-coloured quinoniminedye complex, which can be measured at 505 nm by aUV-spectrophotometer. The intensity of the colour formed is directly proportional to the amount of glucose present in the blood serum.

Total Glucose (mg dL^−1^) = Absorbance of Test/Absorbance of Standard × 100

##### 4.12. Histological Study of Gonads

Gonads were aseptically collected from fish for histological analysis using a modified protocol adapted from [73]. Tissues were fixed in Blouin’s solution (glacial acetic acid, 5%; formaldehyde, 9%; and picric acid, 0.9%) at a 1:10 *w*/*v* ratio for 48 h. Following fixation, tissues were processed using an automated tissue processor and were embedded in paraffin blocks using a histocenter (Thermo-Scientific, Shandon Histocentre, White Marsh, MD, USA). Sections (5–6 μm) were cut using a rotary microtome, stained with haematoxylin and eosin, and mounted with DPX. Slides were examined under a Leica DM 750 microscope at 40× magnification.

##### 4.13. Data Analysis

All the data were subjected to a one-way analysis of variance (ANOVA) using SPSS Version 25.0 software for Windows. Tukey’s multiple comparisons post hoc test was chosen for comparison analysis between the treatments, and a significance level of 5% was adopted. Before running the test, data were subjected to Shapiro–Wilk and Levene’s test to address the normality and homogeneity of variances. Based on growth performance parameters, the optimum concentration GnRH and DOM was ascertained using a broken line regression model. All data are expressed as means ± standard error (S.E.).

## Figures and Tables

**Table 1:** Influence trends of GnRH and DOM supplementation via gel feed on the female GSI of *Devario devario* during the experimental period.

Parameters	Experimental Groups				
	T1 (100 µg GnRH + 50 mg DOM)	T2 (50 µg GnRH + 25 mg DOM)	T3 (25 µg GnRH + 12.5 mg DOM)	T4 (12.5 µg GnRH + 6.25 mg DOM)	Control (0 µg GnRH + 0 mg DOM)
GSI % (initial)	0.4 ± 0.05	0.4 ± 0.05	0.4 ± 0.05	0.4 ± 0.05	0.4 ± 0.05
GSI % (20th)	7.72 ± 0.264 ^c^	6.78 ± 0.546 ^bc^	6.05 ± 0.226 ^b^	4.61 ± 0.205 ^a^	4.33 ± 0.0195 ^a^
GSI % (40th)	2.8 ± 0.741 ^a^	15.2 ± 0.638 ^c^	2.2 ± 0.864 ^a^	9.77 ± 0.159 ^b^	9.49 ± 0.059 ^b^

Results are presented as means ± S.E. of three replicates (*n* = 3). Different superscript letters (a, b, c…) in each row show significant differences among treatments according to Tukey’s test (*p* < 0.05).

**Table 2:** Influence trends of GnRH and DOM supplementation via gel feed on the male GSI of *Devario devario* trend during the experimental period.

Parameters	Experimental Groups				
	T1 (100 µg GnRH + 50 mg DOM)	T2 (50 µg GnRH + 25 mg DOM)	T3 (25 µg GnRH + 12.5 mg DOM)	T4 (12.5 µg GnRH + 6.25 mg DOM)	Control (0 µg GnRH + 0 mg DOM)
GSI % (initial)	0.2 ± 0.05	0.2 ± 0.05	0.2 ± 0.05	0.2 ± 0.05	0.2 ± 0.05
GSI % (20th)	0.779 ± 0.00458 ^c^	0.837 ± 0.0405 ^c^	0.643 ± 0.0186 ^b^	0.643 ± 0.024 ^b^	0.623 ± 0.0231 ^b^
GSI % (40th)	2.62 ± 0.292 ^c^	2.7 ± 0.156 ^c^	3.16 ± 0.191 ^c^	1.19 ± 0.166 ^b^	1.25 ± 0.168 ^b^

Values are presented as means ± S.E. of three replicates (*n* = 3). Different superscript letters (b, c…) in each row show significant differences among treatments according to Tukey’s test (*p* < 0.05).

**Table 3:** Influence of GnRH and DOM supplementation via gel feed on breeding performance of Bengal danio during the experimental period.

Parameters	Experimental Groups				
	T1 (100 µg GnRH + 50 mg DOM)	T2 (50 µg GnRH + 25 mg DOM)	T3 (25 µg GnRH + 12.5 mg DOM)	T4 (12.5 µg GnRH + 6.25 mg DOM)	Control (0 µg GnRH + 0 mg DOM)
Absolute fecundity	1100 ± 36.3 ^b^	1300 ± 23 ^c^	1040 ± 27.5 ^b^	934 ± 39.1 ^a^	936 ± 25 ^a^
Fertilization rate (%)	75.7 ± 0.882 ^a^	76.3 ± 1.45 ^a^	74.3 ± 0.882 ^a^	85.3 ± 0.882 ^b^	86 ± 0.577 ^b^
Hatching percentage (%)	68.7 ± 2.4 ^b^	73.7 ± 1.45 ^c^	68.3 ± 1.76 ^b^	63.7 ± 0.882 ^ab^	62.7 ± 0.882 ^a^
Survival (%)	18.6 ± 0.176 ^a^	18.7 ± 0.0882 ^a^	18.6 ± 0.0577 ^a^	18.6 ± 0.115 ^a^	18.5 ± 0.115 ^a^

Values are presented as means ± S.E. of three replicates (*n* = 3). Different superscript letters (a, b, c…) in each row show significant differences among treatments according to Tukey’s test (*p* < 0.05).

**Table 4:** Observations of sperm quality and quantity of Bengal danio fed with different levels of GnRH and DOM supplementation via gel feed.

Parameters	Experimental Groups				
	T1 (100 µg GnRH + 50 mg DOM)	T2 (50 µg GnRH + 25 mg DOM)	T3 (25 µg GnRH + 12.5 mg DOM)	T4 (12.5 µg GnRH + 6.25 mg DOM)	Control (0 µg GnRH + 0 mg DOM)
Sperm volume (µm^3^)	15.2 ± 0.0577 ^b^	15.4 ± 0.0577 ^b^	15.6 ± 0.153 ^b^	7.37 ± 0.0882 ^a^	7.1 ± 0.115 ^a^
Sperm concentration (10^7^ cell/mL)	3.76 ± 0.0636 ^bc^	4.08 ± 0.0917 ^cd^	4.34 ± 0.159 ^d^	3.47 ± 0.12 ^b^	2.45 ± 0.00882 ^a^
Sperm motility (%)	85.7 ± 0.882 ^a^	86 ± 0.577 ^a^	86 ± 0.577 ^a^	85.7 ± 0.882 ^a^	86 ± 0.577 ^a^

Values are presented as means ± S.E. of three replicates (*n* = 3). Different superscript letters (a, b, c, d) in each row show significant differences among treatments according toTukey’s test (*p* < 0.05).

**Table 5:** Status of cortisol in liver and muscle (ng L^−1^) of *Devario devario* fed with different levels of GnRH- and DOM-supplemented diets.

Parameters	Experimental Groups				
	T1 (100 µg GnRH + 50 mg DOM)	T2 (50 µg GnRH + 25 mg DOM)	T3 (25 µg GnRH + 12.5 mg DOM)	T4 (12.5 µg GnRH + 6.25 mg DOM)	Control (0 µg GnRH + 0 mg DOM)
Liver cortisol (20th)	1.55 ± 0.0186 ^d^	1.35 ± 0.0184 ^c^	1.32 ± 0.00318 ^c^	1.22 ± 0.0104 ^b^	1.15 ± 0.00231 ^a^
Liver cortisol (40th)	0.913 ± 0.0145 ^c^	0.913 ± 0.00882 ^c^	0.88 ± 0.00577 ^bc^	0.857 ± 0.0176 ^ab^	0.837 ± 0.00333 ^a^
Muscle cortisol (20th)	2.46 ± 0.414 ^c^	2.07 ± 0.0124 ^bc^	1.62 ± 0.0569 ^ab^	1.35 ± 0.0427 ^a^	1.28 ± 0.0388 ^a^
Muscle cortisol (40th)	0.973 ± 0.0145 ^d^	0.947 ± 0.012 ^cd^	0.927 ± 0.00882 ^bc^	0.893 ± 0.00882 ^ab^	0.867 ± 0.00882 ^a^

Values are presented as means ± S.E. of three replicates (*n* = 3). Different superscript letters (a, b, c, d) in each row show significant differences among treatments according toTukey’s test (*p* < 0.05).

**Table 6:** Status of glucose in liver and muscle (mg dL^−1^) of *Devario devario* fed with different levels of GnRH- and DOM-supplemented diets.

Parameters	Experimental Groups				
	T1 (100 µg GnRH + 50 mg DOM)	T2 (50 µg GnRH + 25 mg DOM)	T3 (25 µg GnRH + 12.5 mg DOM)	T4 (12.5 µg GnRH + 6.25 mg DOM)	Control (0 µg GnRH + 0 mg DOM)
Liver glucose (20th day)	522 ± 1.62 ^d^	489 ± 0.254 ^c^	434 ± 3.55 ^b^	429 ± 3.33 ^b^	357 ± 3.97 ^a^
Liver glucose (40th day)	365 ± 1.55 ^c^	362 ± 1.86 ^c^	357 ± 1.21 ^b^	355 ± 1.19 ^b^	345 ± 1.24 ^a^
Muscle glucose (20th day)	539 ± 10.2 ^c^	518 ± 10.8 ^c^	467 ± 2.79 ^b^	387 ± 4.1 ^a^	379 ± 7.86 ^a^
Muscle glucose (40th day)	393 ± 0.109 ^b^	391 ± 1.6 ^b^	389 ± 3.34 ^b^	382 ± 0.468 ^a^	377 ± 1.24 ^a^

Values are presented as means ± S.E. of three replicates (*n* = 3). Different superscript letters (a, b, c, d) in each row show significant differences among treatments according to Tukey’s test (*p* < 0.05).

**Table 7:** Status of antioxidant enzymes (SOD and CAT) in brain, gill, liver, and muscle of *Devario devario* fed with different levels of GnRH- and DOM-supplemented diets.

Parameters		Experimental Groups				
		T1 (100 µg GnRH + 50 mg DOM)	T2 (50 µg GnRH + 25 mg DOM)	T3 (25 µg GnRH + 12.5 mg DOM)	T4 (12.5 µg GnRH + 6.25 mg DOM)	Control (0 µg GnRH + 0 mg DOM)
SOD (unit mg protein^−1^)	Brain	35.2 ± 2.44	37.5 ± 2.57	37.9 ± 2.64	46.3 ± 2.15	35.7 ± 2.5
	Gill	73.7 ± 1.83 ^b^	94.2 ± 2.45 ^c^	54.8 ± 2.38 ^a^	57.5 ± 2.51 ^a^	52.3 ± 2.06 ^a^
	Liver	24.6 ± 2.35 ^b^	34.4 ± 2.04 ^c^	12.5 ± 1.98 ^a^	17.6 ± 2.44 ^a^	12.4 ± 2.28 ^a^
	Muscle	41.6 ± 2.05 ^b^	44.6 ± 2.47 ^b^	40.6 ± 2.25 ^b^	30.6 ± 2.28 ^a^	29.7 ± 2.34 ^a^
Catalase (unit mg protein^−1^)	Brain	184 ± 9.36 ^c^	281 ± 24.1 ^d^	145 ± 9.36 ^c^	92.5 ± 3.7 ^b^	40.1 ± 10 ^a^
	Gill	134 ± 17.7 ^c^	281 ± 19.8 ^d^	67.2 ± 7.0 ^bc^	55.5 ± 12 ^bc^	50.1 ± 5.78 ^b^
	Liver	191 ± 6.13 ^c^	265 ± 3.96 ^d^	142 ± 3.54 ^b^	133 ± 6.41 ^b^	60.1 ± 10 ^a^
	Muscle	173 ± 3.54 ^c^	265 ± 14.3 ^d^	149 ± 6.13 ^c^	152 ± 3.7 ^c^	73.4 ± 8.83 ^b^

Values are presented as means ± S.E. of three replicates (*n* = 3). Different superscript letters (a, b, c…) in each row show significant differences among treatments according to Tukey’s test (*p* < 0.05).

**Table 8:** Levels of sex hormones (testosterone and vitellogenin) observed in *Devario devario* fed with different levels of GnRH- and DOM-supplemented diets.

Parameters	Experimental Group				
	T1 (100 µg GnRH + 50 mg DOM)	T2 (50 µg GnRH + 25 mg DOM)	T3 (25 µg GnRH + 12.5 mg DOM)	T4 (12.5 µg GnRH + 6.25 mg DOM)	Control (0 µg GnRH + 0 mg DOM)
Testosterone (nmoL^−1^)	16.1 ± 0.0897 ^c^	16.4 ± 0.255 ^cd^	16.6 ± 0.0176 ^d^	11.1 ± 0.103 ^b^	4.53 ± 0.0841 ^a^
Vitellogenin (nmoL^−1^)	183 ± 6.49 ^c^	189 ± 1.56 ^c^	144 ± 0.583 ^b^	138 ± 1.2 ^ab^	128 ± 14.81 ^a^

Values are presented as means ± S.E. of three replicates (*n* = 3). Different superscript letters (a, b, c, d) in each row show significant differences among treatments according toTukey’s test (*p* < 0.05).

**Table 9:** Nutritional composition of experimental diets (g/kg).

Ingredients	Quantity (g)
Fish muscle	75
Corn flour	30
Salt	2
Lactogen	5
Gelling agent	5
Yeast	2

**Table 10:** Proximate composition (%) of the prepared diets.

Parameters	Percentage	
	Wet wt. Basis	Dry wt. Basis
Moisture	59.79%	---
Protein	22.63%	56.27%
Lipid	5%	12.43%
Ash	8.35%	20.76%
NFE	4.23%	10.54%

NFE = nitrogen-free extract.

**Table 11:** Ranges of different water quality parameters in treatment groups during the experimental period.

Parameters	T1	T2	T3	T4	Control
pH	6.9–7.16	6.8–7.15	6.8–7.12	6.5–7.09	6.5–7.15
Temperature (°C)	26–27.2	25.4–26.3	26.5–27.5	26.4–27.3	26.1–26.9
Dissolved Oxygen (mg L^−1^)	5.1–7.31	5.04–7.2	5.21–7.09	5.3–7.4	5.0–7.2
Total Hardness (mg L^−1^)	51.2–64.4	52.8–63.2	51.7–63.1	53.9–64.1	50.1–63.2
Total Alkalinity (mg L^−1^)	61–79.3	63.3–78.7	61.8–78.1	62.4–77.6	60.2–75

## Data Availability

The data presented in this study are available on request from the corresponding author.
